# Krüppel-Like Transcription Factor KLF1 Is Required for Optimal γ- and β-Globin Expression in Human Fetal Erythroblasts

**DOI:** 10.1371/journal.pone.0146802

**Published:** 2016-02-03

**Authors:** Divya S. Vinjamur, Yousef N. Alhashem, Safa F. Mohamad, Parth Amin, David C. Williams, Joyce A. Lloyd

**Affiliations:** 1 Department of Human and Molecular Genetics, Virginia Commonwealth University, Richmond, Virginia, United States of America; 2 Massey Cancer Center, Virginia Commonwealth University, Richmond, Virginia, United States of America; 3 Department of Pathology, Virginia Commonwealth University, Richmond, Virginia, United States of America; Southern Illinois University School of Medicine, UNITED STATES

## Abstract

In human adult erythroid cells, lower than normal levels of Krüppel-like transcription factor 1 (KLF1) are generally associated with decreased adult β- and increased fetal γ-globin gene expression. KLF1 also regulates BCL11A, a known repressor of adult γ-globin expression. In seeming contrast to the findings in adult cells, lower amounts of KLF1 correlate with both reduced embryonic and reduced fetal β-like globin mRNA in mouse embryonic erythroid cells. The role of KLF1 in primary human fetal erythroid cells, which express both γ- and β-globin mRNA, is less well understood. Therefore, we studied the role of KLF1 in *ex vivo* differentiated CD34+ umbilical cord blood cells (UCB erythroblasts), representing the fetal milieu. In UCB erythroblasts, KLF1 binds to the β-globin locus control region (LCR), and the β-globin promoter. There is very little KLF1 binding detectable at the γ-globin promoter. Correspondingly, when cultured fetal UCB erythroblasts are subjected to lentiviral KLF1 knockdown, the active histone mark H3K4me3 and RNA pol II recruitment are diminished at the β- but not the γ-globin gene. The amount of KLF1 expression strongly positively correlates with β-globin mRNA and weakly positively correlates with BCL11A mRNA. With modest KLF1 knockdown, mimicking haploinsufficiency, γ-globin mRNA is increased in UCB erythroblasts, as is common in adult cells. However, a threshold level of KLF1 is evidently required, or there is no absolute increase in γ-globin mRNA in UCB erythroblasts. Therefore, the role of KLF1 in γ-globin regulation in fetal erythroblasts is complex, with both positive and negative facets. Furthermore, in UCB erythroblasts, diminished BCL11A is not sufficient to induce γ-globin in the absence of KLF1. These findings have implications for the manipulation of BCL11A and/or KLF1 to induce γ-globin for therapy of the β-hemoglobinopathies.

## Introduction

Krüppel-like factor 1 (KLF1 or EKLF, Erythroid Krüppel-like factor) is a transcription factor that regulates erythrocyte development, including β-like globin gene expression, and synthesis of heme, the cytoskeleton, and the cell membrane ([1–3] reviewed in [4]). KLF1-/- mouse embryos exhibit fatal β-thalassemia [5,6]. In the mouse, KLF1 binds to the β-globin locus control region (LCR) and gene promoters, and interacts with histone acetyl transferases (HATs) [7–9]. Furthermore, KLF1 is necessary for normal chromatin looping between the β-globin LCR and promoters [10], and co-association of KLF1-regulated genes in nuclear transcription factories [11].

The role of KLF1 in fetal-to-adult globin gene switching is currently studied in human systems, because reactivation of γ-globin has therapeutic potential for the β-hemoglobinopathies. Naturally occurring heterozygous KLF1 mutations can cause hereditary persistence of fetal hemoglobin (α2γ2) [12–14]. In fact, heterozygous KLF1 mutations are relatively more common in a thalassemia endemic region in China, and apparently ameliorate the severity of β-thalassemia by increasing HbF [15]. Recently, there was a case report of an individual who is KLF1 null. This patient required transfusions from the age of 6 months due to anemia, but displayed very elevated fetal hemoglobin (HbF) of >70% up to 1 year of age [14]. In the laboratory, lentiviral knockdown of KLF1 in cultured adult erythroblasts results in increased γ-globin and decreased BCL11A, a γ-globin repressor [12,16–18].

The mode of action of KLF1 in globin switching includes direct regulation of the BCL11A gene [12,17]. The negative role of KLF1 in γ-globin gene expression in adult (definitive) human erythroid cells contrasts with its positive role in mouse embryonic (primitive) erythroid cells. In mouse embryonic erythroblasts, KLF1 has a positive effect on embryonic and γ-globin gene expression [19]. Because of these contrasting functions of KLF1 in human adult compared to mouse embryonic erythroid cells, we wished to determine the role of KLF1 in γ- to β-globin switching in primary human fetal erythroblasts. In fetal definitive erythroblasts, we find that the effects of KLF1 on the γ-globin gene can be both negative and positive.

## Materials and Methods

### Plasmid construction

Two KLF1-targeted shRNA (shRNA-V1 and shRNA-V2) and scramble (Scr) shRNA vectors, also encoding green fluorescence protein (GFP), were acquired from Drs. Morlé and Louache [20]. These vectors were constructed on a pRRL backbone (Bouilloux, Blood 2008) with the shRNA-coding sequence under the control of the H1 promoter. Although KLF1 and KLF2 have similar sequences, the KLF1 shRNAs are KLF1-specific.

### Lentiviral preparation

The pRRL lentiviral plasmids were packaged into lentiviruses in 293T cells using the pCMV-dR8.74 packaging plasmid and pMD2G envelope plasmid via calcium phosphate transfections [21]. Virus containing media were harvested to transduce CD34+ cells.

### CD34+ cell isolation, culture and transduction

Fresh umbilical cord blood (collected within 24–48 hours) was obtained from the St. Louis Cord Blood Bank (SLCBB, St. Louis, MO). GE Ficoll Paque™ Premium (GE Healthcare) was used to isolate mononuclear cells following the manufacturer’s protocol. CD34+ cells were enriched from the mononuclear cells by magnetic separation using the EasySep™ human CD34 positive selection kit (Stem Cell Technologies). CD34+ cells were expanded for 8 days in StemSpan SFEM medium (Stem Cell Technologies) containing 1X CC100 cytokine cocktail (Stem Cell Technologies), 8 μl/ml LDL (Sigma, L7914) and 2% Penicillin/Streptomycin (P/S, Gibco) [22]. The lentivirus-containing media were used to transduce CD34+ cells on expansion day 8. On the third day after infection, successfully transduced GFP+ cells were collected by FACS and differentiated along the erythroid lineage [22,23]. UCB erythroblast pellets were red when harvested on differentiation day 8 (DD8).

### ChIP assays

Chromatin immunoprecipitation (ChIP) assays were performed essentially as described previously [9,24], or using the Magna ChIP kit (EMD Millipore). The antibodies for ChIP are listed in the S1 Table of the Supporting Information. Purified DNA was quantitated using quantitative PCR (qPCR) and SYBR Green chemistry. Fold enrichment was calculated as 2^(Ct_input_−Ct_test_) and expressed relative to the IgG control. The oligonucleotide primer sequences used for qPCR are in the S2 Table.

### Quantitative reverse transcriptase-PCR (qRT-PCR)

Changes in gene expression were measured by quantitative reverse transcriptase-PCR (qRT-PCR) using cDNA prepared with the iScript cDNA synthesis kit (Biorad). qRT-PCR was performed using SYBR Green or Taqman reagent (Applied Biosystems) on an ABI Prism 7900HT analyzer (Applied Biosystems) [9]. The oligonucleotide primer sequences used for qRT-PCR are in the S2 Table. The relative quantification method (E^Ct_endogenous gene_/E^Ct_test gene_ where E = efficiency of primer set) was employed to analyze changes in gene expression in KLF1-shRNA treated samples compared to Scr-shRNA controls. The mRNA amount in Scr shRNA-treated control cells was considered 100%, and fold change for KLF1 shRNA-treated cells was calculated by intra-sample comparisons. A standard curve was run for each gene and used to calculate the efficiency of the respective primer set.

### Statistical analyses

The Student’s t-test or Pearson’s correlation were used for statistical analyses. *P*<0.05 was considered significant.

## Results and Discussion

**Fig 1:**
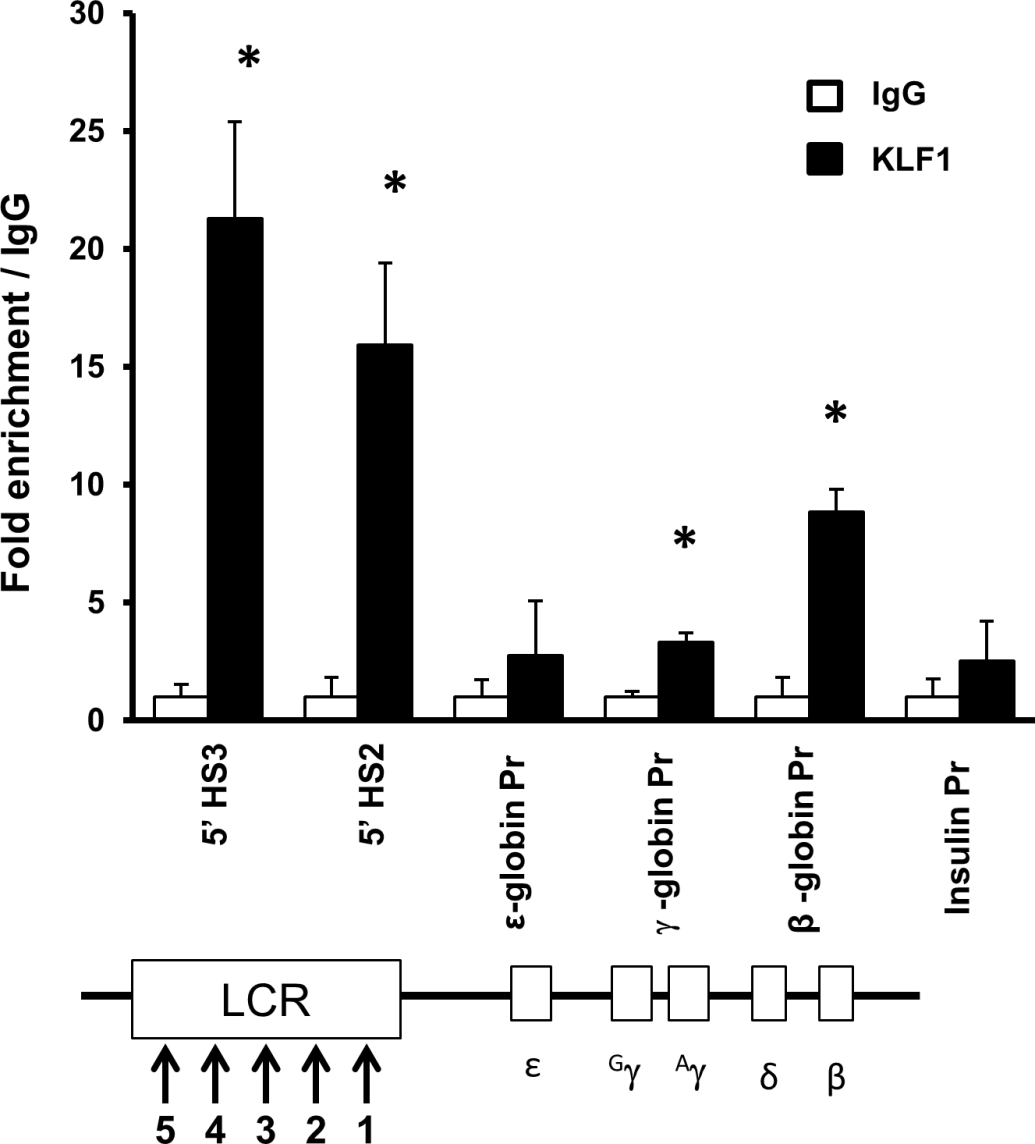
KLF1 is enriched at the locus control region (LCR) and β-globin promoter in umbilical cord blood (UCB) erythroblasts. CD34+ cells were enriched from umbilical cord blood units, expanded for eight days, and *in vitro* differentiated for eight days (DD8). Chromatin immunoprecipitation (ChIP) assays were performed with DD8 erythroblasts. Chromatin was incubated with either a KLF1 antibody or non-specific antibody (IgG) as a negative control (black or white bars, respectively). Precipitated chromatin was subjected to quantitative PCR (qPCR) to determine the amount of precipitated DNA from 5’HS3 and 5’HS2 in the LCR, and from the ε-, γ- and β-globin promoters. The primers for the γ-globin promoter collectively measure the ^A^γ- and ^G^γ-globin genes. The insulin promoter was used as a negative control. Pr = promoter, N = 2, error bars = standard error, * = p-value<0.05 compared to non-specific antibodies. β-globin locus not drawn to scale.

Umbilical cord blood (UCB) erythroblasts express similar amounts of fetal γ- and adult β-globin mRNA (S1A Fig), representative of a fetal milieu. The cells express increasing amounts of globin and KLF1 mRNA over an 8 day differentiation period (S1A and S1B Fig). ChIP assays indicate that KLF1 is enriched in the β-globin locus control region (LCR) at 5’ DNase I hypersensitive sites, 5’HS3 and 5’HS2, and at the β-globin promoter in UCB erythroblasts at differentiation day 8 (DD8) (Fig 1). A limited amount of KLF1 binding is detectable at the γ-globin promoter. These results suggest that KLF1 directly regulates the β-globin gene, but probably has less direct impact on expression of the γ-globin gene in UCB erythroblasts.

**Fig 2:**
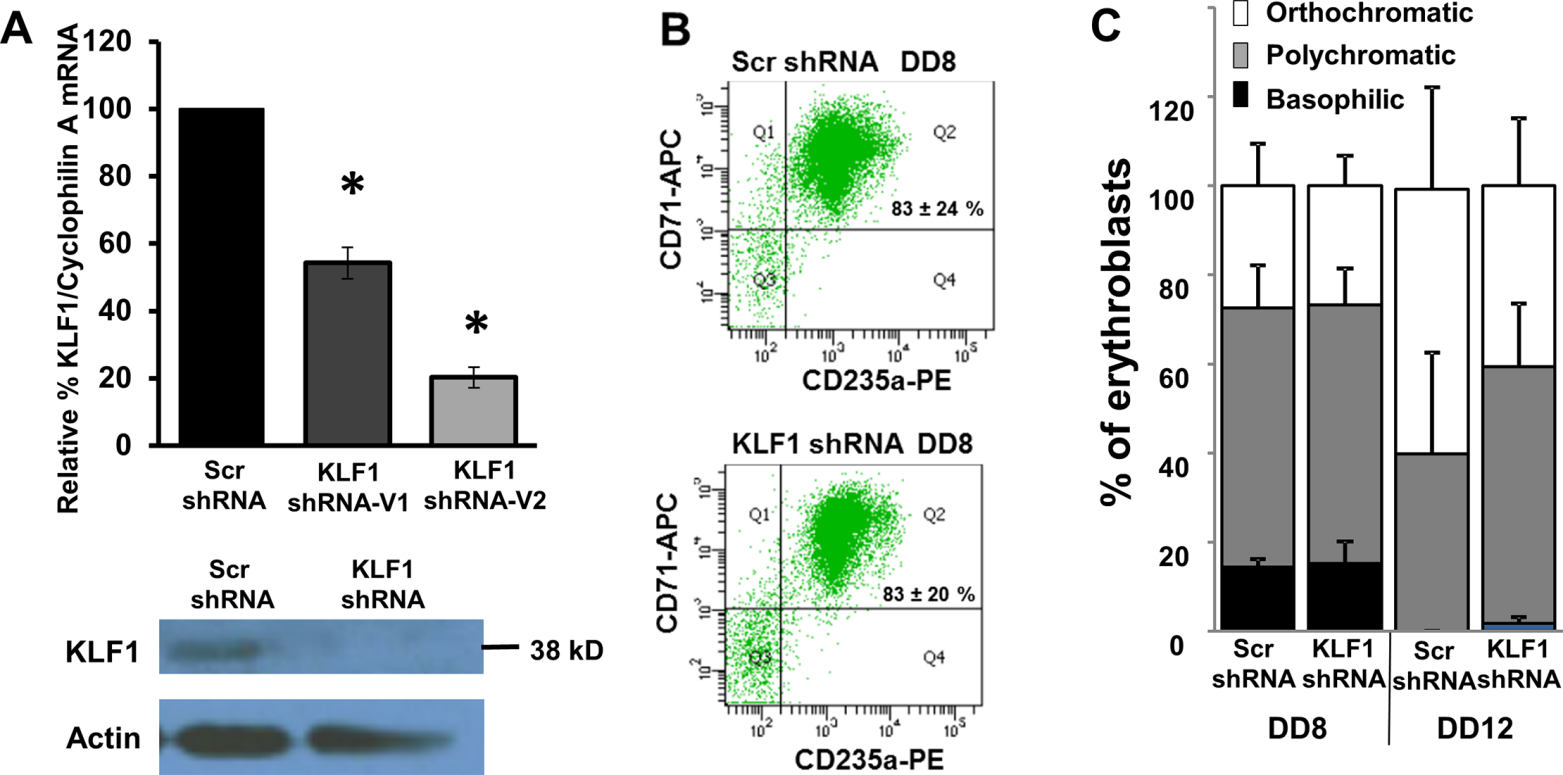
Scr and KLF1 shRNA-treated UCB erythroblasts have similar erythroid differentiation profiles on differentiation day 8 (DD8). **(A) Efficiency of KLF1 knockdown.** Top panel: KLF1 mRNA amount was measured in scramble (Scr) and KLF1 shRNA-treated cells by qRT-PCR. The amount of KLF1 mRNA in scramble-treated cells was set to 100 for each sample. Cyclophilin A mRNA was used as the internal standard for qRT-PCR. N = 19 for Scr shRNA, N = 3 for KLF1 shRNA-V1 and N = 16 for KLF1 shRNA-V2; error bars = standard error, * = p-value<0.001. Bottom panel: Representative Western blot analysis in Scr and KLF1 shRNA treated UCB erythroblasts. Actin was used as a loading control. For this sample, residual KLF1 mRNA = 13% and residual KLF1 protein = 42%. **(B) KLF1 knockdown does not adversely affect erythroid differentiation.** Representative flow cytometry plots indicate that the majority of erythroid cells are double positive for CD71 and CD235a in both scramble shRNA-infected and KLF1 shRNA-V2-infected cells on DD8. N = 3. Student’s t-test showed no significant difference in the number of double positive cells between Scr and KLF1 shRNA-treatment groups. Residual KLF1 mRNA = 13–25%. **(C) KLF1 knockdown does not affect erythroid maturation.** At day 8 and at day 12 of differentiation, cytospin slides of Scr or KLF1 shRNA treated cells were prepared, and erythroid maturation was scored blind to treatment group. 100 cells from each slide were scored based on their morphology and designated as basophilic, polychromatic or orthochromatic erythroblasts. N = 5, error bars = standard error. Student’s t-test showed no significant differences in the number of erythroblasts of each type between Scr and KLF1 shRNA treatment groups. Residual KLF1 mRNA = 6–24%.

Lentiviral KLF1 knockdown was achieved at the RNA and protein level in UCB erythroblasts (Fig 2A). Multiple lines of evidence indicate that cells with robust KLF1 knockdown differentiate normally to DD8. On average, more than 80% of cells infected with either the control Scr or KLF1 shRNA are double positive for transferrin receptor and Glycophorin A (CD71^+^CD235a^+^), which indicates erythroid differentiation (Fig 2B). DD8 cells are hemoglobinized, as evidenced by a red cell pellet and positive benzidine staining. Cytospins of DD8 erythroblasts show identical erythroid maturation in Scr and KLF1 shRNA transduced cells (Fig 2C), as is the case for bone marrow-derived erythroblasts [17]. At DD12, the erythroblasts are more differentiated than at DD8, but DD12 cells with the KLF1 shRNA appear to trend towards being less mature than those with the Scr shRNA (Fig 2C). In order to focus the study on the roles of KLF1 in globin gene expression, without confounding effects on cellular differentiation due to KLF1 knockdown, the remaining experiments were performed with DD8 erythroblasts. It is predicted that at later time points, differentiation of UCB cells with KLF1 knockdown would become progressively more severely disrupted, because a KLF1 null patient is severely anemic [14].

**Fig 3:**
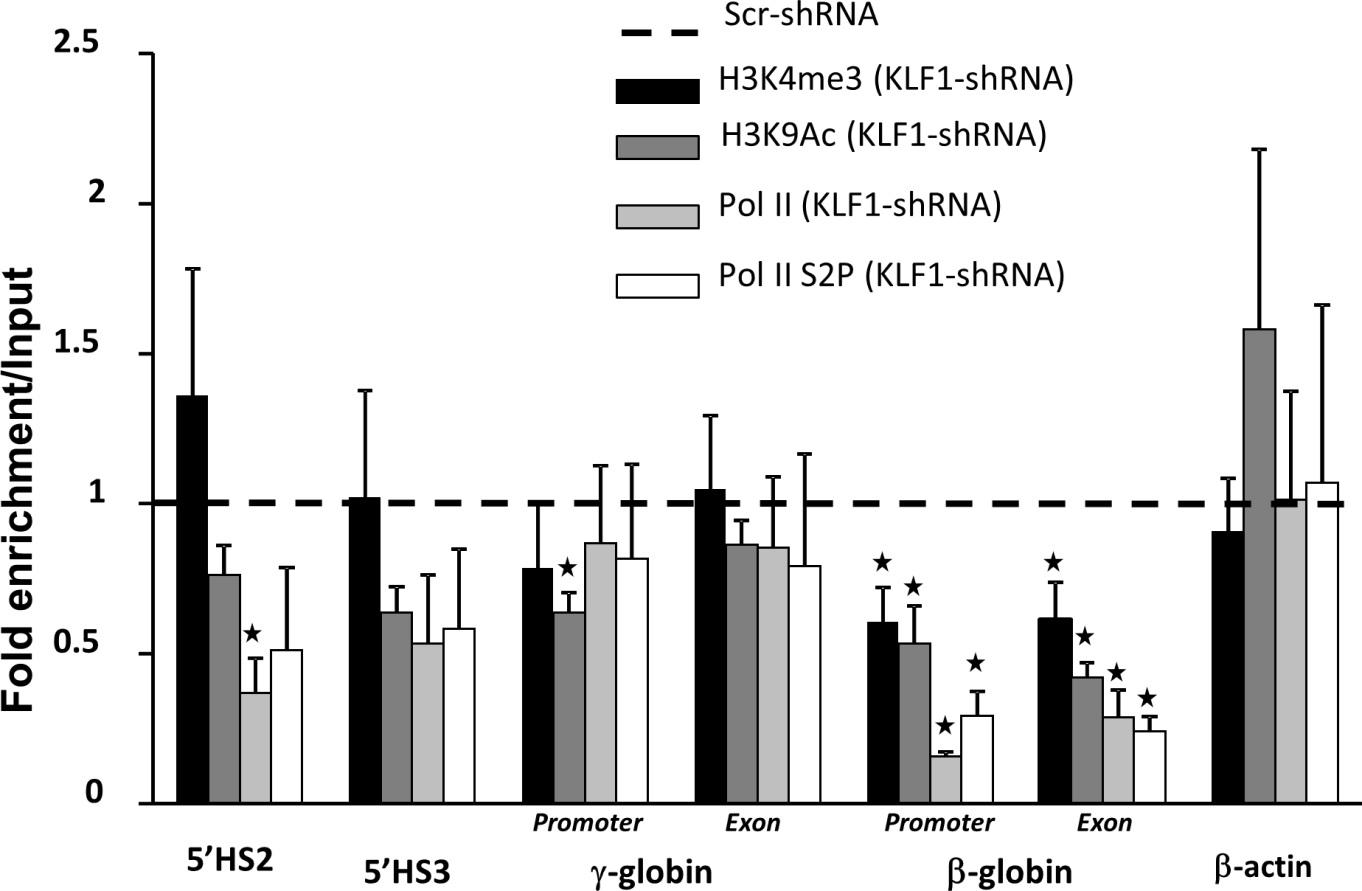
KLF1 knockdown reduces H3K4me3 and RNA polymerase II recruitment at the β- but not γ-globin gene. ChIP assays were performed using anti-H3K9Ac, anti-H3K4me3, anti-RNA Pol II or anti-RNA Pol II S2P antibodies on Scr and KLF1 shRNA-treated UCB erythroblasts. H3K9 acetylation generally indicates open chromatin configuration and H3K4 trimethylation marks regions of active transcription. qPCR was used to quantify enrichment at 5’HS3, 5’HS2 and at sites within the promoter and exon 2 of the γ- and β-globin genes. The primers for the γ-globin promoter and exon collectively measure the ^A^γ- and ^G^γ-globin genes. Non-specific antibodies (IgG) were used as a control for antibody specificity. β-actin was used as a control gene not regulated by KLF1. All regions of the globin locus that were tested show significant enrichment for H3K9Ac, H3K4me3, RNA Pol II and RNA Pol II-S2P compared to IgG in normal (Scr-shRNA treated) cells (data not shown). Enrichment in Scr shRNA-treated cells was set to 1 and relative enrichment in KLF1 shRNA-treated cells is shown for each genomic region tested. N = 4, error bars = standard error, * = p-value<0.05 compared to Scr shRNA. Residual KLF1 mRNA = 11–31% in KLF1 shRNA-treated samples.

Ablation of KLF1 in transgenic mouse embryos with the human β-globin locus results in diminished active histone marks, histone 3 lysine 9 acetylation (H3K9Ac) and histone 3 lysine 4 trimethylation (H3K4me3) at the ε- and γ-globin genes [9]. KLF1 knockdown in UCB erythroblasts results in significantly reduced H3K9Ac and H3K4me3 at the β-globin gene and promoter, but H3K4me3 is not reduced at the γ-globin gene and promoter, 5’HS2 or 5’HS3 (Fig 3). Similarly, recruitment of RNA polymerase II (Pol II) and the active form of Pol II (Pol II phospho-serine 2, Pol II S2P) are reduced at the β- but not γ-globin gene with KLF1 knockdown (Fig 3). These results further suggest that KLF1 has direct effects on the regulation of the β- but not the γ-globin gene.

**Fig 4:**
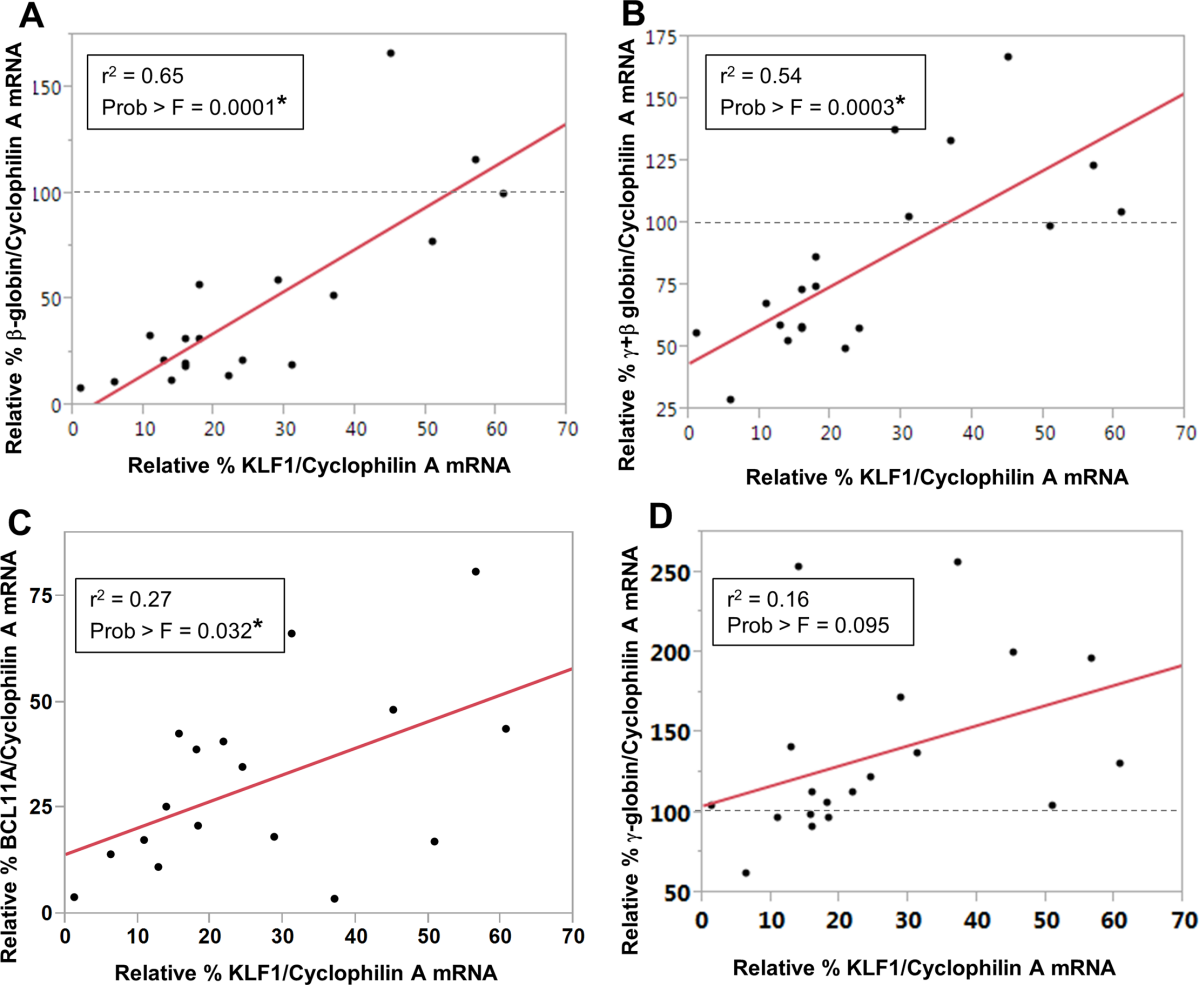
KLF1 regulates β-globin, total globin and BCL11A expression in UCB erythroblasts. The amount of KLF1, β-globin, γ-globin and BCL11A mRNA was measured by qRT-PCR and normalized to Cyclophilin A mRNA. The fold change in expression of these genes in KLF1 shRNA-treated samples was calculated by setting the value in Scr shRNA controls to 100. Each point on the Pearson’s correlation plots represents a biological replicate, i.e. cells obtained from a different umbilical cord blood sample. Three of the samples were infected with the K1V1 shRNA, and the remaining samples (the majority) were infected with the K1V2 shRNA. **(A) A strong, positive correlation was observed between the amounts of KLF1 and β-globin mRNA** (N = 19, r^2^ = 0.65, Prob>F = 0.0001); **(B) The amount of total globin (γ+β) expression positively correlates with residual KLF1 mRNA** (N = 19, r^2^ = 0.54, Prob>F = 0.0003) **(C) There is a modest positive correlation between the amounts of KLF1 and BCL11A mRNA** (N = 17, r^2^ = 0.27, Prob>F = 0.032). **(D) There is no linear relationship between the amounts of KLF1 and γ-globin mRNA** (N = 19, r^2^ = 0.16, Prob>F = 0.095).

KLF1 knockdown resulted in decreased expression of β-globin mRNA in UCB erythroblasts (Fig 4A), as expected from the reduction in active chromatin marks (Fig 3), and consistent with adult cells [5,6,12,17]. Using a large number of samples with a range of KLF1 knockdown efficiencies, it was possible to determine that β-globin expression level correlates positively and significantly with the residual amount of KLF1 mRNA (r^2^ = 0.65, Prob>F = 0.0001). The y-intercept of the line predicts that there would be virtually no β-globin RNA in UCB erythroblasts without KLF1, as expected, because KLF1 is indispensable for β-globin expression. Fig 4B indicates that there is also a significant and strong positive correlation between KLF1 and total globin (γ+β) mRNA, although the y-intercept predicts approximately 40% total globin even without any KLF1, presumably due to the continued expression of γ-globin mRNA (discussed below). BCL11A mRNA is decreased with KLF1 knockdown, and there is a weak but significant positive correlation between KLF1 and BCL11A expression (r^2^ = 0.27, Prob>F = 0.032) (Fig 4C). Human UCB erythroblasts show a small decrease in α-globin mRNA with KLF1 knockdown (S2A Fig), as previously determined for mouse fetal cells [5,6].

**Fig 5:**
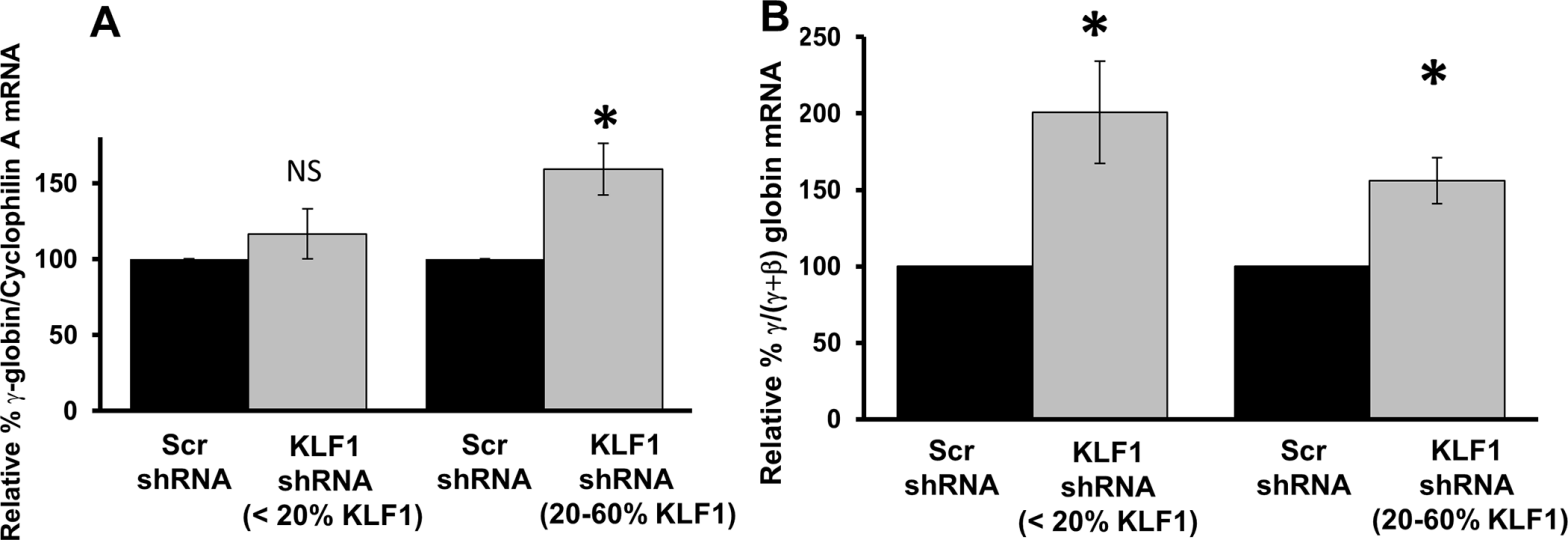
γ-globin mRNA amounts with modest and robust KLF1 knockdown. UCB samples were divided into two groups based on the amount of residual KLF1 mRNA after knockdown (see supplementary Fig 2B). The first group consists of samples which showed robust KLF1 knockdown (<20% residual KLF1; N = 10). The second group had modest knockdown of KLF1 (20–60% residual KLF1, N = 9). The primers for γ-globin collectively measure ^A^γ- and ^G^γ-globin mRNA. **A) Absolute amounts of γ-globin mRNA are increased above normal only in the presence of a threshold amount of KLF1.** Within each group, the fold change in γ-globin expression in KLF1 shRNA-treated cells was calculated by setting the amount of γ-globin mRNA in Scr shRNA-treated controls to 100. Cyclophilin A mRNA was used as the internal standard for qPCR. Error bars = standard error, * = p-value<0.005, NS = not significant. **(B) γ-globin is increased relative to total (γ+β)-globin with any amount of KLF1 knockdown.** The amount of γ-globin mRNA in Scr and KLF1 shRNA-treated cells was measured by qRT-PCR and reported as a fraction of total (γ+β)-globin mRNA. In each group the fold change in γ-globin expression in KLF1 shRNA-treated cells was calculated by setting the amount of γ-globin in Scr shRNA-treated controls to 100. Error bars = standard error, * = p-value<0.01.

Surprisingly, there is no linear relationship between KLF1 and γ-globin expression in UCB erythroblasts, using the Pearson’s correlation in the same manner (r^2^ = 0.16, Prob>F = 0.095) (Fig 4D). A smooth curve fit to the data suggests that the relationship between KLF1 and γ-globin RNA amounts may depend on the efficiency of KLF1 knockdown (S2B Fig). With modest KLF1 knockdown, defined as 20–60% residual KLF1 mRNA, there is a significant 1.5 to 2-fold increase in γ-globin expression compared to Scr shRNA controls (Fig 5A, right). Logically, the fold-induction of γ-globin is about 2-fold lower in fetal than in adult cells [18], because γ-globin is more abundant in fetal cells. With robust KLF1 knockdown, or <20% residual KLF1 mRNA, the absolute amount of γ-globin expression is normal, i.e. not increased compared to Scr shRNA transduced cells (Fig 5A, left), even though there is very little BCL11A mRNA (<25%, see Fig 4C). These results are similar to those obtained from KLF1 knockout mouse embryos, though in that case, γ-globin mRNA is actually reduced compared to normal when KLF1 is completely ablated [9]. In the UCB erythroblasts, γ-globin mRNA is not more labile due to KLF1 knockdown; there is no difference in the ratio of nascent to mature γ-globin RNA in Scr compared to KLF1 shRNA-treated cells (unpublished data). Although absolute γ-globin mRNA amounts are near normal in UCB erythroblasts with <20% residual KLF1 mRNA, if γ-globin is expressed as a percentage of total globin, as is often done in the literature, it appears to be increased (Fig 5B). However, this apparent increase in the ratio is due solely to the decrease in β-globin mRNA in the denominator (see Fig 4A).

These findings suggest that although KLF1 is not required for γ-globin expression in human UCB erythroblasts, a threshold amount of KLF1 is required to achieve induction of γ-globin above normal levels. This is a novel finding from this study. Our results indicate that with almost complete ablation of KLF1, UCB erythroblasts produce normal amounts of γ-globin mRNA, but reduced β-globin and reduced total globin mRNA. Our data are consistent with the case report of the KLF1 null individual, who had >70% HbF, but had low total hemoglobin and was transfusion dependent [14]. The UCB samples with intermediate knockdown efficiency (20–60%) had increased γ-globin mRNA amounts of 150–200%, which is consistent with some patients with heterozygous KLF1 mutations, who tend to have elevated fetal hemoglobin.

There are two possible reasons why the requirement for a threshold amount of KLF1 in order to achieve γ-globin induction has not previously been reported. The KLF1 knockdown efficiency achieved in this study with UCB erythroblasts may exceed that described previously in cultured adult erythroblasts, or the large number of samples examined here may have facilitated the observation. Alternatively, the requirement for KLF1 to achieve γ-globin induction may be unique to fetal cells and not apply to adult cells. Even in this case, the results suggest that treatments involving KLF1 inhibition may work differently in children and adults with β-hemoglobinopathies.

Because KLF2 is highly homologous to KLF1, and KLF2 can regulate globin gene expression [9,19,25], KLF2 mRNA amounts were measured in human UCB erythroblasts subjected to KLF1 knockdown. As shown in the S3 Fig, there is increased KLF2 mRNA concomitant with KLF1 knockdown. However, the relationship between the expression levels of the two KLF genes is complex, and non-linear. It is possible that KLF2 can partially compensate for KLF1 to allow γ-globin induction when KLF1 knockdown is 20–60%, but not when KLF1 knockdown is more efficient.

Then again, there are other possible reasons for the absence of γ-globin induction in cells with efficient KLF1 knockdown. For example, sufficient KLF1 binding within the β-globin locus is required to establish chromatin architecture [10], and interactions between the LCR and γ-globin gene may be required for induction. Furthermore, the absence of KLF1 could lead to reduced expression of other transcription factors, which are in turn required for γ-globin induction. It should be noted that these putative mechanisms are not mutually exclusive, and other more complex scenarios are also possible.

Our data complement and extend other recent work, indicating that KLF1, BCL11A, and γ-globin do not have simple linear relationships in fetal and adult cells. Esteghamat et al. [26] found that human β-globin locus mice with compound mutations in the KLF1 and BCL11A genes do not sustain high levels of γ-globin as adults. Trakarnsanga et al. showed that ectopic expression of both KLF1 and the longest form of BCL11A are required to induce β-globin expression in induced pluripotent stem cells and UCB erythroblasts [27]. Grieco et al. have recently shown that down-regulation of a transcription factor network including KLF1, BCL11A and TAL1 accompanies induction of γ-globin expression with hydroxyurea, a drug which is used to treat β-hemoglobinopathies [28]. Our data indicate that diminished KLF1 results in reduced BCL11A expression, but highly efficient KLF1 knockdown does not result in an increase in γ-globin mRNA in fetal cells.

These findings provide evidence that should be considered in developing strategies to knockdown KLF1 as a means to induce γ-globin, as a treatment for the hemoglobinopathies. KLF1 is an attractive therapeutic target, because it is erythroid-specific, and persons with heterozygous KLF1 mutations are not seriously ill. The results in this work further suggest that it may be beneficial to knockdown KLF1 by approximately half, and not much more, in therapeutic strategies to induce fetal hemoglobin.

## Conclusions

In conclusion, there are two main findings in this work. KLF1 mRNA amount clearly dictates the β-globin expression level in primary human umbilical cord blood (UCB)-derived erythroblasts. KLF1 mRNA amount is not strictly correlated with γ-globin mRNA amount. Modest knockdown of KLF1 correlates with induced γ-globin expression, but a threshold level of KLF1 is required for this activation of γ-globin expression in UCB-derived erythroblasts.

## Supporting Information

S1 Fig Gene expression during erythroid differentiation of CD34+ umbilical cord blood cells. Cells were collected for RNA extraction on days 0, 2, 4, 6 and 8 of erythroid differentiation. On differentiation day 8 (DD8), pelleted cells are bright red and stain positively for hemoglobin using benzidine (data not shown). Cyclophilin A was used as a normalization control for qRT-PCR. N = 3, Error bars = standard error. **(A) γ- and β-globin mRNA are expressed in UCB erythroblasts.** Globin mRNA was measured using qRT-PCR and expressed using the E^ΔCT^ method to allow for direct comparison of γ- and β-globin mRNA amounts. ε-globin mRNA is present at only negligible levels in UCB erythroblasts (>60-fold lower than γ- or β-globin). **(B) KLF1 expression during differentiation of UCB erythroblasts.** The amount of KLF1 mRNA was measured using qRT-PCR.
(PPTX)

S2 Fig Role of KLF1 in α- and γ-globin gene expression. **(A) KLF1 positively regulates α-globin expression.** α-globin mRNA amount was measured in Scr and KLF1 shRNA-treated cells by qRT-PCR. The amount of α-globin mRNA in Scr shRNA- treated cells was set to 100 for each sample. Cyclophilin A mRNA was used as the internal standard for qRT-PCR. N = 8; error bars = standard error, * = p-value<0.05. **(B) KLF1 regulation of γ-globin expression.** γ-globin mRNA amount was measured in Scr and KLF1 shRNA-treated cells by qRT-PCR. The amount of γ-globin mRNA in Scr shRNA-treated cells was set to 100% for each sample. Cyclophilin A mRNA was used as the internal standard for qRT-PCR. N = 19. This smooth curve is the best fit generated using JMP software. By definition, at 100% KLF1 mRNA there is 100% γ-globin mRNA.
(PPTX)

S3 Fig Role of KLF1 in KLF2 gene expression. KLF2 mRNA amount was measured in Scr and KLF1 shRNA-treated cells by qRT-PCR. The amount of KLF2 mRNA in Scr shRNA-treated cells was set to 100% for each sample. Cyclophilin A mRNA was used as the internal standard for qRT-PCR. N = 18. **(A) There is no linear relationship between the amounts of KLF1 and KLF2 mRNA** (r^2^ = 0.13, Prob>F = 0.148). **(B) Smooth curve for best fit of KLF2 mRNA with KLF1 knockdown.** In most samples, KLF2 mRNA is increased with KLF1 knockdown. The curve was generated using JMP software.
(PPTX)

S1 Table Antibodies for ChIP and Western blotting. The antibodies used for ChIP and western blotting are listed, including the company from which they were purchased (source) and the catalog numbers.
(PPTX)

S2 Table Oligonucleotide primers used for ChIP qPCR and qRT-PCR. The sequences of the oligonucleotide primers used for qPCR are listed from 5’ to 3’. F is the forward primer and R is the reverse primer. The primers for ChIP-qPCR were used on genomic DNA templates, and the primers for qRT-PCR were used on cDNA templates. The sequences of additional qRT-PCR primers were published previously [9].
(PPTX)
