# Supplementary material for: Krüppel-Like Transcription Factor KLF1 Is Required for Optimal γ- and β-Globin Expression in Human Fetal Erythroblasts
Source: PLoS One. 2016 Feb 3;11(2):e0146802. doi: 10.1371/journal.pone.0146802 (PMC4739742; doi:10.1371/journal.pone.0146802)
Supplement: S1 Fig — Cells were collected for RNA extraction on days 0, 2, 4, 6 and 8 of erythroid differentiation. On differentiation day 8 (DD8), pelleted cells are bright red and stain positively for hemoglobin using benzidine (data not shown). Cyclophilin A was used as a normalization control for qRT-PCR. N = 3, Error bars = standard error. (A) γ- and β-globin mRNA are expressed in UCB erythroblasts. Globin mRNA was measured using qRT-PCR and expressed using the EΔCT method to allow for direct comparison of γ- and β-globin mRNA amounts. ε-globin mRNA is present at only negligible levels in UCB erythroblasts (>60-fold lower than γ- or β-globin). (B) KLF1 expression during differentiation of UCB erythroblasts. The amount of KLF1 mRNA was measured using qRT-PCR. (PPTX) [file pone.0146802.s001.pptx]

## Slide 1
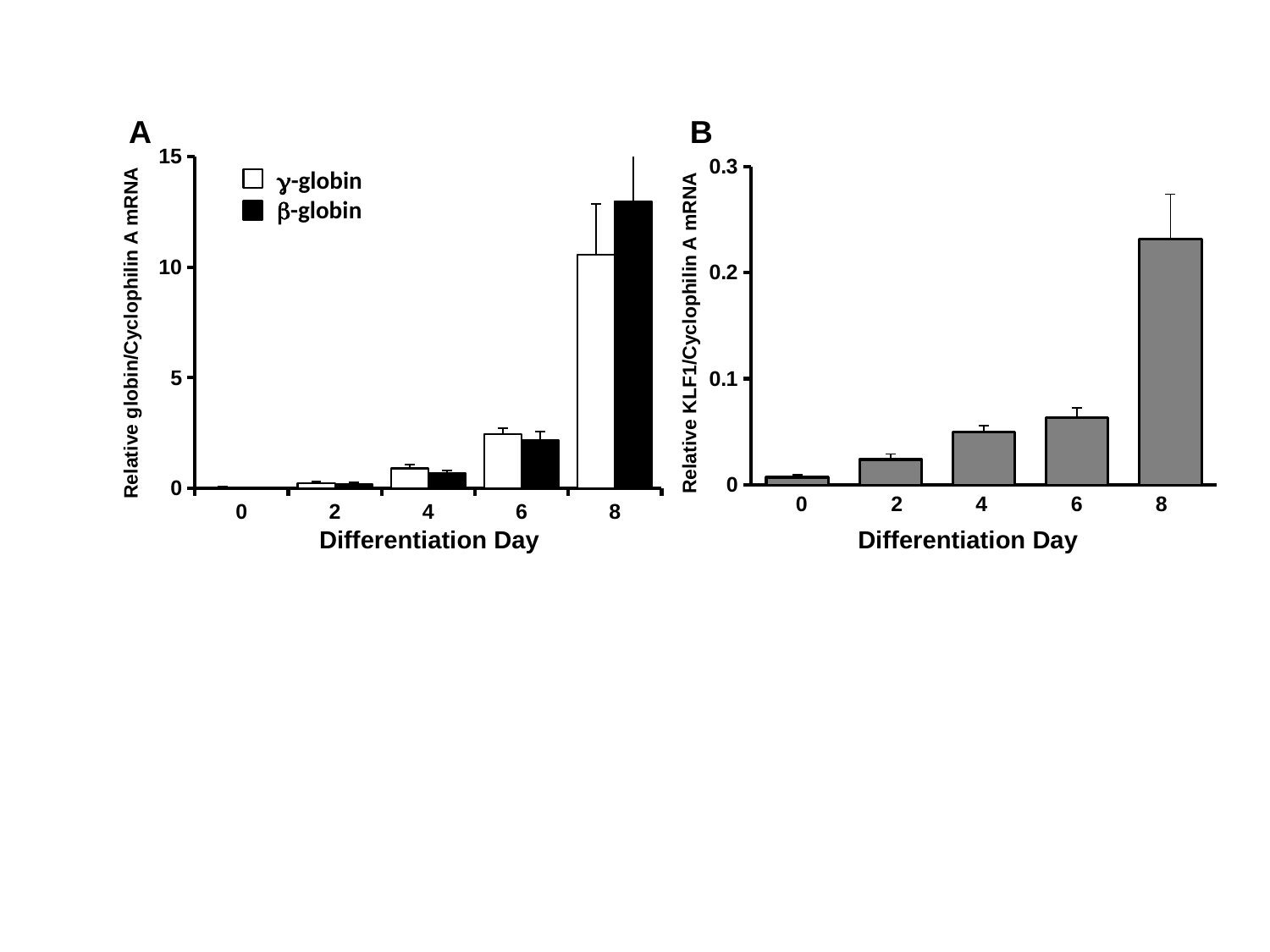

A
Relative globin/Cyclophilin A mRNA
Differentiation Day
### Chart
| Category | γ-Globin | β-Globin |
|---|---|---|
| 0 | 0.0434 | 0.0345 |
| 2 | 0.2304 | 0.2033 |
| 4 | 0.8939 | 0.6842 |
| 6 | 2.435999999999998 | 2.1757 |
| 8 | 10.5562 | 12.9775 |g-globin
b-globin
B
### Chart
| Category | |
|---|---|Relative KLF1/Cyclophilin A mRNA
0
2
4
6
8
Differentiation Day
