# Supplementary material for: Krüppel-Like Transcription Factor KLF1 Is Required for Optimal γ- and β-Globin Expression in Human Fetal Erythroblasts
Source: PLoS One. 2016 Feb 3;11(2):e0146802. doi: 10.1371/journal.pone.0146802 (PMC4739742; doi:10.1371/journal.pone.0146802)
Supplement: S2 Fig — (A) KLF1 positively regulates α-globin expression. α-globin mRNA amount was measured in Scr and KLF1 shRNA-treated cells by qRT-PCR. The amount of α-globin mRNA in Scr shRNA- treated cells was set to 100 for each sample. Cyclophilin A mRNA was used as the internal standard for qRT-PCR. N = 8; error bars = standard error, * = p-value<0.05. (B) KLF1 regulation of γ-globin expression. γ-globin mRNA amount was measured in Scr and KLF1 shRNA-treated cells by qRT-PCR. The amount of γ-globin mRNA in Scr shRNA-treated cells was set to 100% for each sample. Cyclophilin A mRNA was used as the internal standard for qRT-PCR. N = 19. This smooth curve is the best fit generated using JMP software. By definition, at 100% KLF1 mRNA there is 100% γ-globin mRNA. (PPTX) [file pone.0146802.s002.pptx]

## Slide 1
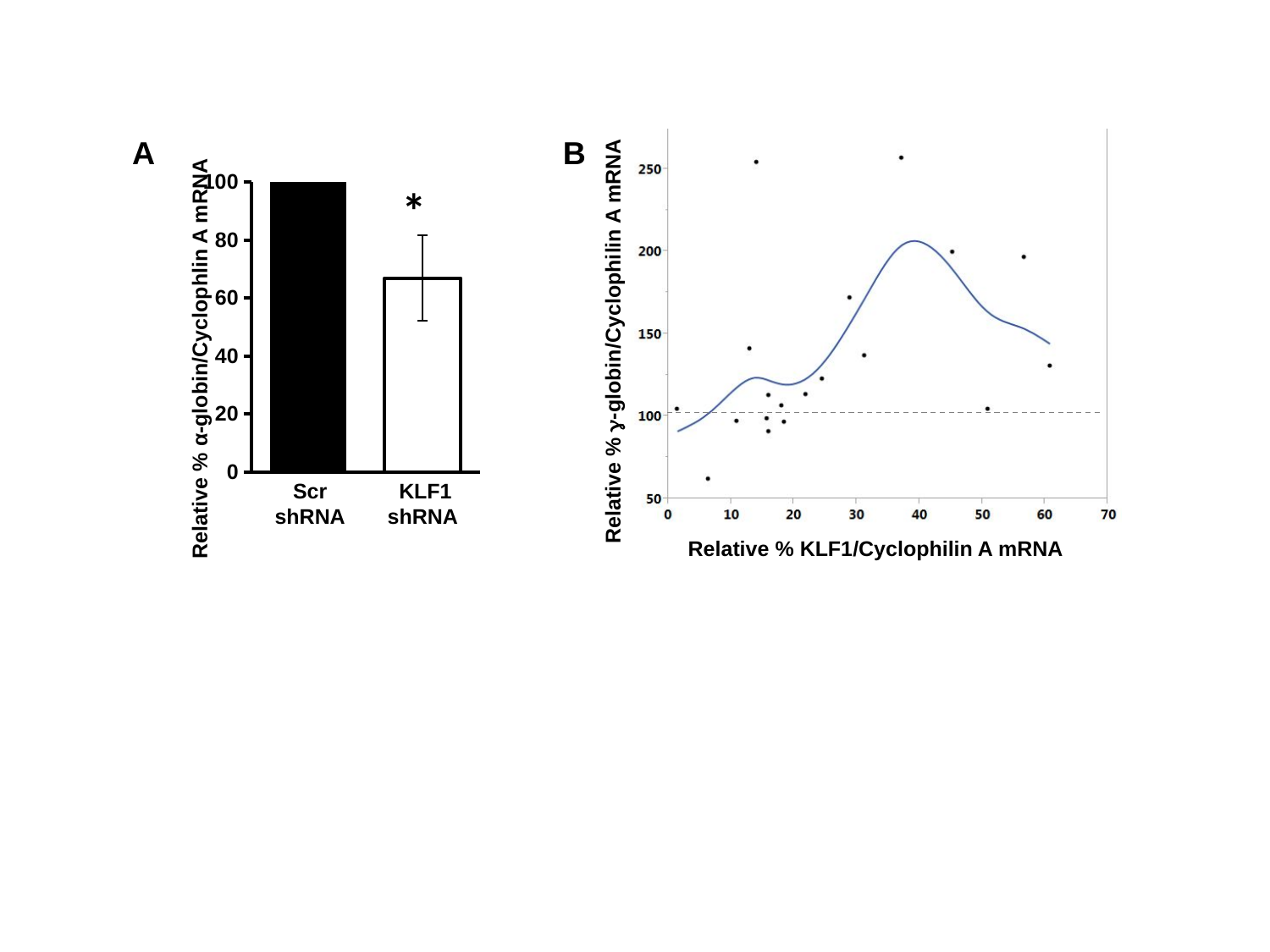

A
B
### Chart
| Category | |
|---|---|*
Relative % α-globin/Cyclophlin A mRNA
Scr shRNA
KLF1 shRNA
Relative % γ-globin/Cyclophilin A mRNA
Relative % KLF1/Cyclophilin A mRNA
