# Supplementary material for: Krüppel-Like Transcription Factor KLF1 Is Required for Optimal γ- and β-Globin Expression in Human Fetal Erythroblasts
Source: PLoS One. 2016 Feb 3;11(2):e0146802. doi: 10.1371/journal.pone.0146802 (PMC4739742; doi:10.1371/journal.pone.0146802)
Supplement: S3 Fig — KLF2 mRNA amount was measured in Scr and KLF1 shRNA-treated cells by qRT-PCR. The amount of KLF2 mRNA in Scr shRNA-treated cells was set to 100% for each sample. Cyclophilin A mRNA was used as the internal standard for qRT-PCR. N = 18. (A) There is no linear relationship between the amounts of KLF1 and KLF2 mRNA (r2 = 0.13, Prob>F = 0.148). (B) Smooth curve for best fit of KLF2 mRNA with KLF1 knockdown. In most samples, KLF2 mRNA is increased with KLF1 knockdown. The curve was generated using JMP software. (PPTX) [file pone.0146802.s003.pptx]

## Slide 1
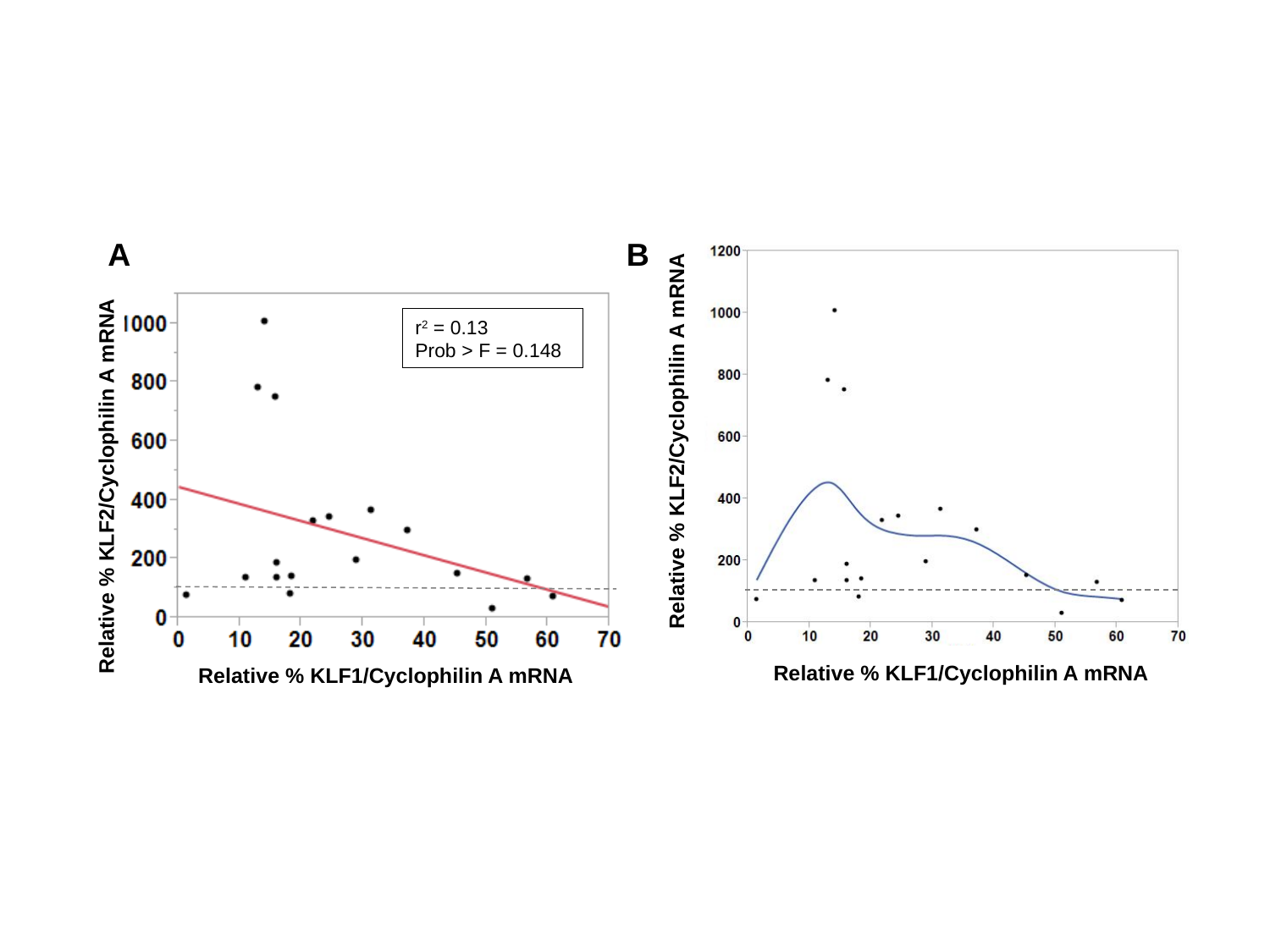

A
B
r2 = 0.13
Prob > F = 0.148
Relative % KLF2/Cyclophilin A mRNA
Relative % KLF2/Cyclophilin A mRNA
Relative % KLF1/Cyclophilin A mRNA
Relative % KLF1/Cyclophilin A mRNA
