# Supplementary material for: Krüppel-Like Transcription Factor KLF1 Is Required for Optimal γ- and β-Globin Expression in Human Fetal Erythroblasts
Source: PLoS One. 2016 Feb 3;11(2):e0146802. doi: 10.1371/journal.pone.0146802 (PMC4739742; doi:10.1371/journal.pone.0146802)
Supplement: S1 Table — The antibodies used for ChIP and western blotting are listed, including the company from which they were purchased (source) and the catalog numbers. (PPTX) [file pone.0146802.s004.pptx]

## Slide 1
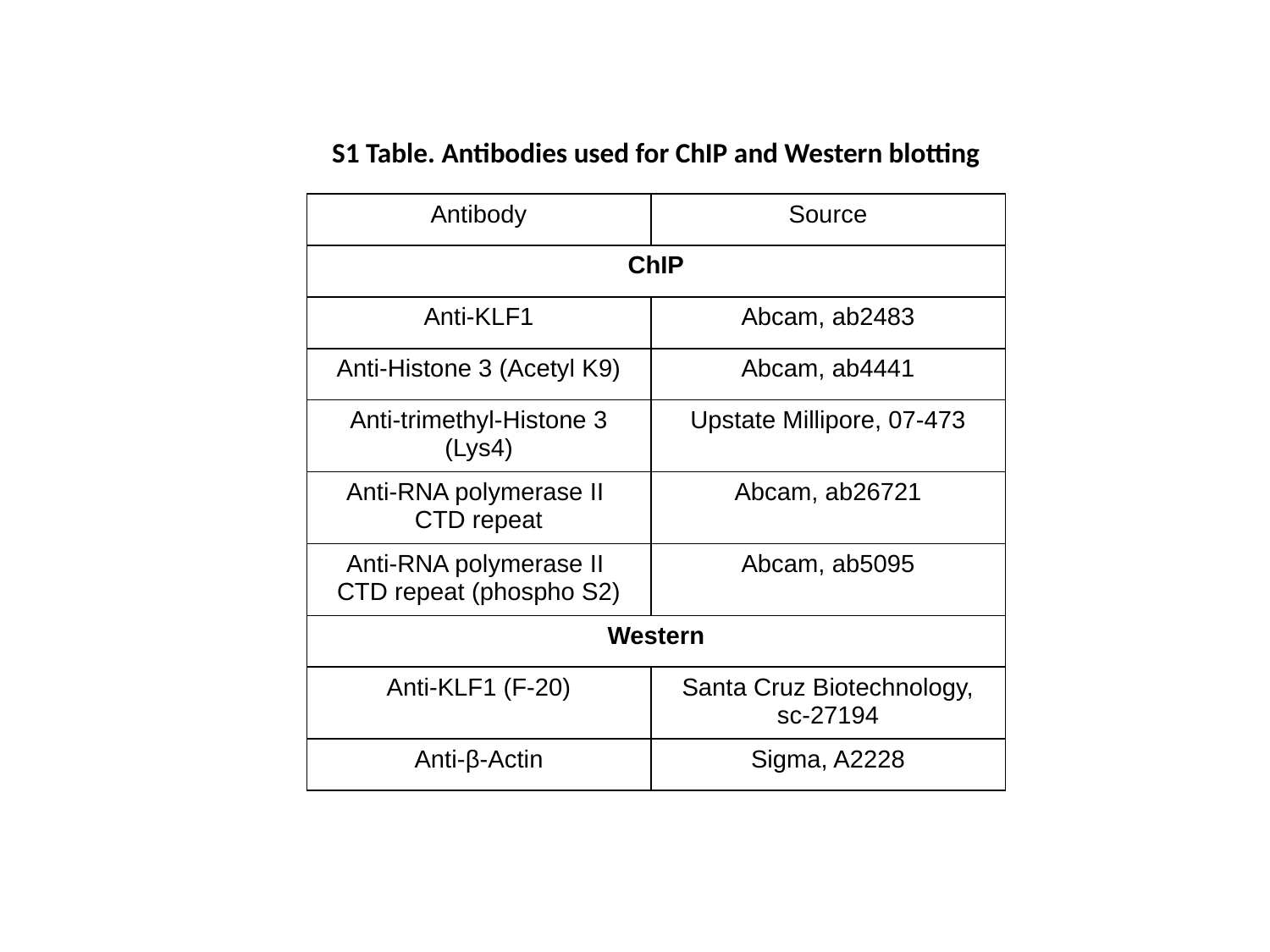

S1 Table. Antibodies used for ChIP and Western blotting
| Antibody | Source |
| --- | --- |
| ChIP | |
| Anti-KLF1 | Abcam, ab2483 |
| Anti-Histone 3 (Acetyl K9) | Abcam, ab4441 |
| Anti-trimethyl-Histone 3 (Lys4) | Upstate Millipore, 07-473 |
| Anti-RNA polymerase II CTD repeat | Abcam, ab26721 |
| Anti-RNA polymerase II CTD repeat (phospho S2) | Abcam, ab5095 |
| Western | |
| Anti-KLF1 (F-20) | Santa Cruz Biotechnology, sc-27194 |
| Anti-β-Actin | Sigma, A2228 |
