# Supplementary material for: Krüppel-Like Transcription Factor KLF1 Is Required for Optimal γ- and β-Globin Expression in Human Fetal Erythroblasts
Source: PLoS One. 2016 Feb 3;11(2):e0146802. doi: 10.1371/journal.pone.0146802 (PMC4739742; doi:10.1371/journal.pone.0146802)
Supplement: S2 Table — The sequences of the oligonucleotide primers used for qPCR are listed from 5’ to 3’. F is the forward primer and R is the reverse primer. The primers for ChIP-qPCR were used on genomic DNA templates, and the primers for qRT-PCR were used on cDNA templates. The sequences of additional qRT-PCR primers were published previously [9]. (PPTX) [file pone.0146802.s005.pptx]

## Slide 1
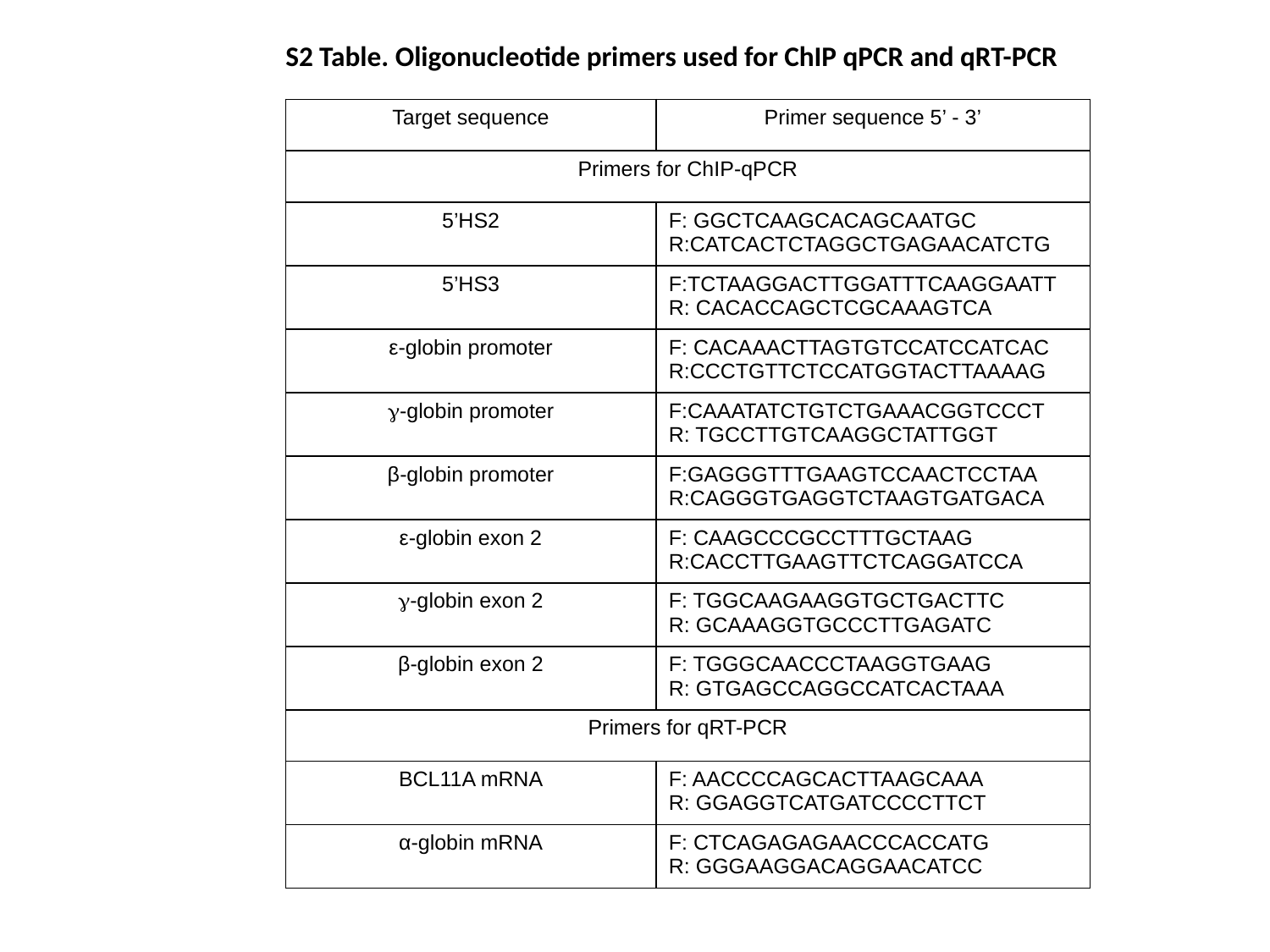

S2 Table. Oligonucleotide primers used for ChIP qPCR and qRT-PCR
| Target sequence | Primer sequence 5’ - 3’ |
| --- | --- |
| Primers for ChIP-qPCR | |
| 5’HS2 | F: GGCTCAAGCACAGCAATGC R:CATCACTCTAGGCTGAGAACATCTG |
| 5’HS3 | F:TCTAAGGACTTGGATTTCAAGGAATT R: CACACCAGCTCGCAAAGTCA |
| ε-globin promoter | F: CACAAACTTAGTGTCCATCCATCAC R:CCCTGTTCTCCATGGTACTTAAAAG |
| γ-globin promoter | F:CAAATATCTGTCTGAAACGGTCCCT R: TGCCTTGTCAAGGCTATTGGT |
| β-globin promoter | F:GAGGGTTTGAAGTCCAACTCCTAA R:CAGGGTGAGGTCTAAGTGATGACA |
| ε-globin exon 2 | F: CAAGCCCGCCTTTGCTAAG R:CACCTTGAAGTTCTCAGGATCCA |
| γ-globin exon 2 | F: TGGCAAGAAGGTGCTGACTTC R: GCAAAGGTGCCCTTGAGATC |
| β-globin exon 2 | F: TGGGCAACCCTAAGGTGAAG R: GTGAGCCAGGCCATCACTAAA |
| Primers for qRT-PCR | |
| BCL11A mRNA | F: AACCCCAGCACTTAAGCAAA R: GGAGGTCATGATCCCCTTCT |
| α-globin mRNA | F: CTCAGAGAGAACCCACCATG R: GGGAAGGACAGGAACATCC |
